# 1,3,5-Triaza­adamantan-7-amine

**DOI:** 10.1107/S1600536810037657

**Published:** 2010-09-25

**Authors:** Jaclyn Thomson, Danielle M. Chisholm, Allen G. Oliver, J. Scott McIndoe

**Affiliations:** a Department of Chemistry, University of Victoria, PO Box 3065, Victoria, BC V8W 3V6, Canada; b University of Notre Dame, Department of Chemistry and Biochemistry, 251 Nieuwland Science Hall, Notre Dame, IN 46556-5670, USA

## Abstract

The title compound, C_7_H_14_N_4_, represents the first structurally characterized, isolated triaza­adamantane. In the crystal structure, weak inter­molecular N—H⋯N hydrogen bonds link the mol­ecules into columns about the crystallographic fourfold axis.

## Related literature

For general background to applications of the title compound and its preparation, see: Hodge (1972 ▶); Karelina *et al.* (1987 ▶); Kuznetsov *et al.* (2001 ▶); Nielsen (1975 ▶, 1977 ▶); Safar *et al.* (1975 ▶). For related structures, see: de Namor *et al.* (2008 ▶).
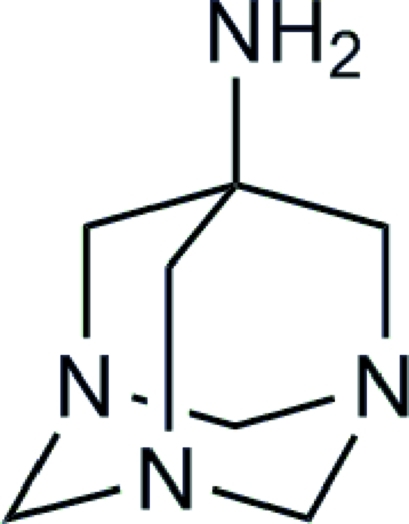


## Experimental

### 

#### Crystal data

C_7_H_14_N_4_
*M* *_r_* = 154.22
Tetragonal, 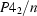

*a* = 15.5402 (8) Å
*c* = 6.5074 (7) Å
*V* = 1571.5 (2) Å^3^
*Z* = 8
Mo *K*α radiation
μ = 0.09 mm^−1^
*T* = 150 K
0.32 × 0.24 × 0.15 mm

#### Data collection

Bruker APEXII diffractometer
Absorption correction: numerical (*SADABS*; Sheldrick, 2008*a* ▶) *T*_min_ = 0.973, *T*_max_ = 0.987
15555 measured reflections
1960 independent reflections
1662 reflections with *I* > 2σ(*I*)
*R*_int_ = 0.032

#### Refinement

*R*[*F* ^2^ > 2σ(*F* ^2^)] = 0.039
*wR*(*F* ^2^) = 0.122
*S* = 1.52
1960 reflections
106 parameters
H atoms treated by a mixture of independent and constrained refinement
Δρ_max_ = 0.28 e Å^−3^
Δρ_min_ = −0.17 e Å^−3^

### 

Data collection: *APEX2* (Bruker, 2005 ▶); cell refinement: *SAINT* (Bruker, 2005 ▶); data reduction: *SAINT*; program(s) used to solve structure: *SHELXS97* (Sheldrick, 2008*b* ▶); program(s) used to refine structure: *SHELXL97* (Sheldrick, 2008*b* ▶); molecular graphics: *XP* (Sheldrick, 2008*b* ▶) and *POV-RAY* (Cason, 2003 ▶); software used to prepare material for publication: *XCIF* (Sheldrick, 2008*b* ▶) and *publCIF* (Westrip, 2010 ▶).

## Supplementary Material

Crystal structure: contains datablocks I, global. DOI: 10.1107/S1600536810037657/cv2767sup1.cif

Structure factors: contains datablocks I. DOI: 10.1107/S1600536810037657/cv2767Isup2.hkl

Additional supplementary materials:  crystallographic information; 3D view; checkCIF report

## Figures and Tables

**Table 1:** Hydrogen-bond geometry (Å, °)

*D*—H⋯*A*	*D*—H	H⋯*A*	*D*⋯*A*	*D*—H⋯*A*
N11—H11*A*⋯N3^i^	0.898 (12)	2.335 (13)	3.2316 (13)	176.0 (12)
N11—H11*B*⋯N11^ii^	0.895 (14)	2.253 (14)	3.1465 (14)	175.2 (11)

Symmetry codes: (i) 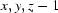; (ii) 
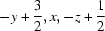
.

**Fig. 1.**
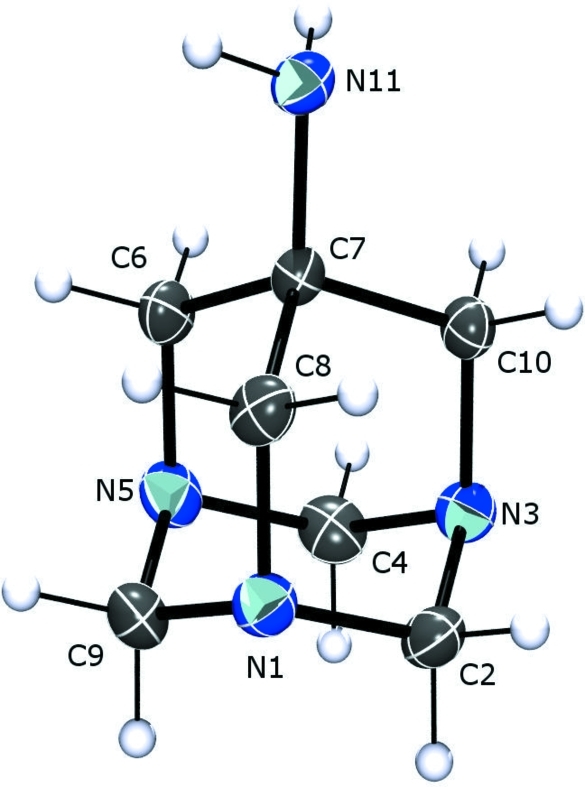
View of 1 showing 50% probability displacement ellipsoids.

**Fig. 2.**
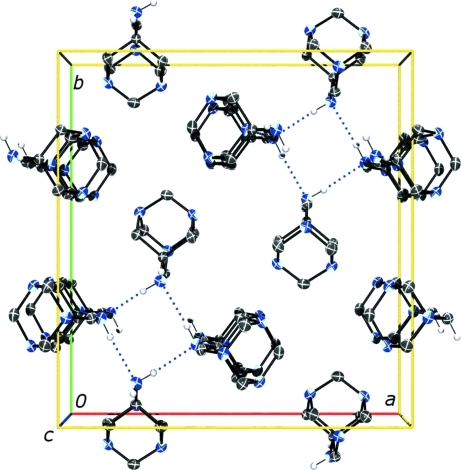
Hydrogen-bonding and packing of 1 viewed along the c-axis. Dotted lines represent hydrogen bonding.

## supplementary crystallographic information

### Comment

The title compound, 7-amino-1,3,5-triazaadamantane, 1, is a member of the class of adamantane compounds. To the best of our knowledge there are only two structurally characterized compounds containing the 1,3,5-triazaadamantane moiety (de Namor *et al.*, 2008). However, both of the previously characterized species incorporate 7-nitro-1,3,5-triazaadamantane and are complexed with mercury.

7-Amino-1,3,5-triazaadamantane has been used as a curing agent in a number of processes from providing an alternative fuel source (Nielsen, 1977) to rubber manufacture (Karelina *et al.*, 1987). Further it has been used as a precursor to other adamantane compounds (notably phosphaazaadamantanes, see for example Kuznetsov *et al.*, 2001). The reactivity of the amino functionality has been investigated widely.

In the solid state the compound forms one-dimensional H-bonded (Table 1) chains that run through the lattice parallel to the crystallographic *c*-axis about the 4_2_ screw-axis (Figure 2). The hydrogen-bonding is only exhibited through contacts from the amino group to neighbouring amino groups. The three N atoms in the azaadamantane portion of the molecule are not involved in any intermolecular contacts.

### Experimental

7-Amino-1,3,5-triazaadamantane was prepared (Safar *et al.*, 1975) by Pd/C/H_2_ reduction of 7-nitro-1,3,5-triazaadamantane (Hodge, 1972) in ethanol. NMR data was consistent with literature values (Nielsen, 1975). Single crystals suitable for X-ray crystallography were obtained from cooling a saturated ethanol solution of 7-amino-1,3,5-triazaadamantane to 4°C for one week.

### Refinement

C-bound H atoms were placed in geometrically idealized positions (C—H = 0.99 Å), and refined as riding with *U*_iso_(H) = 1.2*U*_eq_(C) . Amino H-atoms were located on a difference map, and refined with bond restraint N—H = 0.90 (1) Å, and constraint *U*_iso_(H) = 1.2*U*_eq_(N).

### Crystal data

**Table:**

C_7_H_14_N_4_	*D*_x_ = 1.304 Mg m^−^^3^
*M**_r_* = 154.22	Mo *K*α radiation, λ = 0.71073 Å
Tetragonal, *P*4_2_/*n*	Cell parameters from 4624 reflections
Hall symbol: -P 4bc	θ = 2.6–28.2°
*a* = 15.5402 (8) Å	µ = 0.09 mm^−^^1^
*c* = 6.5074 (7) Å	*T* = 150 K
*V* = 1571.5 (2) Å^3^	Columnar, colourless
*Z* = 8	0.32 × 0.24 × 0.15 mm
*F*(000) = 672	

### Data collection

**Table:**

Bruker APEXII diffractometer	1960 independent reflections
Radiation source: fine-focus sealed tube	1662 reflections with *I* > 2σ(*I*)
graphite	*R*_int_ = 0.032
Detector resolution: 83.33 pixels mm^-1^	θ_max_ = 28.3°, θ_min_ = 1.9°
ω/2θ–scans	*h* = −20→20
Absorption correction: numerical (*SADABS*; Sheldrick, 2008a)	*k* = −19→20
*T*_min_ = 0.973, *T*_max_ = 0.987	*l* = −8→8
15555 measured reflections	

### Refinement

**Table:**

Refinement on *F*^2^	Primary atom site location: structure-invariant direct methods
Least-squares matrix: full	Secondary atom site location: difference Fourier map
*R*[*F*^2^ > 2σ(*F*^2^)] = 0.039	Hydrogen site location: inferred from neighbouring sites
*wR*(*F*^2^) = 0.122	H atoms treated by a mixture of independent and constrained refinement
*S* = 1.52	*w* = 1/[σ^2^(*F*_o_^2^) + (0.056*P*)^2^] where *P* = (*F*_o_^2^ + 2*F*_c_^2^)/3
1960 reflections	(Δ/σ)_max_ = 0.035
106 parameters	Δρ_max_ = 0.28 e Å^−^^3^
0 restraints	Δρ_min_ = −0.17 e Å^−^^3^

### Special details

**Table:**

Geometry. All e.s.d.'s (except the e.s.d. in the dihedral angle between two l.s. planes) are estimated using the full covariance matrix. The cell e.s.d.'s are taken into account individually in the estimation of e.s.d.'s in distances, angles and torsion angles; correlations between e.s.d.'s in cell parameters are only used when they are defined by crystal symmetry. An approximate (isotropic) treatment of cell e.s.d.'s is used for estimating e.s.d.'s involving l.s. planes.
Refinement. Refinement of *F*^2^ against ALL reflections. The weighted *R*-factor *wR* and goodness of fit *S* are based on *F*^2^, conventional *R*-factors *R* are based on *F*, with *F* set to zero for negative *F*^2^. The threshold expression of *F*^2^ > σ(*F*^2^) is used only for calculating *R*-factors(gt) *etc*. and is not relevant to the choice of reflections for refinement. *R*-factors based on *F*^2^ are statistically about twice as large as those based on *F*, and *R*- factors based on ALL data will be even larger.The amino H atoms were located from a difference Fourier map and included with refined coordinates and thermal parameters tied to that of N11. All other H atoms were included in geometrically calculated positions with thermal parameters tied to that of the carbon to which they are bonded.

### Fractional atomic coordinates and isotropic or equivalent isotropic displacement parameters (Å^2^)

**Table:**

	*x*	*y*	*z*	*U*_iso_*/*U*_eq_	
N1	0.62365 (6)	0.43256 (6)	0.53674 (13)	0.0254 (2)	
C2	0.62425 (7)	0.48052 (7)	0.73108 (16)	0.0269 (3)	
H2A	0.6191	0.4393	0.8464	0.032*	
H2B	0.5734	0.5189	0.7352	0.032*	
N3	0.70245 (6)	0.53264 (6)	0.76040 (12)	0.0251 (2)	
C4	0.77644 (8)	0.47390 (7)	0.75022 (15)	0.0304 (3)	
H4A	0.7729	0.4328	0.8661	0.037*	
H4B	0.8300	0.5077	0.7673	0.037*	
N5	0.78167 (6)	0.42511 (6)	0.55650 (14)	0.0283 (2)	
C6	0.78851 (7)	0.48753 (7)	0.38591 (16)	0.0251 (2)	
H6A	0.7917	0.4560	0.2539	0.030*	
H6B	0.8422	0.5212	0.4014	0.030*	
C7	0.71110 (6)	0.54920 (6)	0.38172 (14)	0.0199 (2)	
C8	0.62901 (7)	0.49473 (7)	0.36610 (16)	0.0243 (2)	
H8A	0.5781	0.5329	0.3692	0.029*	
H8B	0.6287	0.4634	0.2337	0.029*	
C9	0.70056 (7)	0.37757 (7)	0.53292 (17)	0.0297 (3)	
H9A	0.6961	0.3346	0.6448	0.036*	
H9B	0.7020	0.3458	0.4010	0.036*	
C10	0.70855 (7)	0.59524 (6)	0.59025 (14)	0.0222 (2)	
H10A	0.7613	0.6303	0.6070	0.027*	
H10B	0.6584	0.6344	0.5947	0.027*	
N11	0.71492 (6)	0.61249 (6)	0.21797 (14)	0.0255 (2)	
H11A	0.7093 (8)	0.5886 (8)	0.0929 (19)	0.031*	
H11B	0.7652 (9)	0.6402 (9)	0.2292 (18)	0.031*	

### Atomic displacement parameters (Å^2^)

**Table:**

	*U*^11^	*U*^22^	*U*^33^	*U*^12^	*U*^13^	*U*^23^
N1	0.0272 (5)	0.0259 (5)	0.0232 (4)	−0.0056 (3)	0.0002 (3)	−0.0014 (3)
C2	0.0300 (6)	0.0293 (6)	0.0213 (5)	−0.0041 (4)	0.0042 (4)	0.0000 (4)
N3	0.0312 (5)	0.0259 (5)	0.0181 (4)	−0.0026 (4)	−0.0029 (3)	0.0000 (3)
C4	0.0318 (6)	0.0313 (6)	0.0282 (6)	0.0007 (5)	−0.0092 (4)	0.0038 (4)
N5	0.0273 (5)	0.0244 (5)	0.0332 (5)	0.0036 (3)	−0.0004 (4)	0.0017 (4)
C6	0.0224 (5)	0.0244 (5)	0.0285 (5)	0.0022 (4)	0.0039 (4)	−0.0012 (4)
C7	0.0200 (5)	0.0217 (5)	0.0179 (5)	0.0001 (4)	0.0004 (3)	−0.0009 (3)
C8	0.0238 (5)	0.0294 (6)	0.0198 (5)	−0.0038 (4)	−0.0027 (4)	−0.0024 (4)
C9	0.0364 (7)	0.0216 (6)	0.0312 (6)	−0.0017 (4)	0.0015 (4)	−0.0014 (4)
C10	0.0249 (5)	0.0216 (5)	0.0202 (5)	−0.0003 (4)	−0.0017 (4)	−0.0019 (4)
N11	0.0300 (5)	0.0274 (5)	0.0191 (4)	−0.0016 (4)	0.0006 (3)	0.0009 (3)

### Geometric parameters (Å, °)

**Table:**

N1—C2	1.4679 (13)	C6—H6A	0.9900
N1—C9	1.4695 (15)	C6—H6B	0.9900
N1—C8	1.4742 (13)	C7—N11	1.4514 (12)
C2—N3	1.4729 (14)	C7—C10	1.5346 (13)
C2—H2A	0.9900	C7—C8	1.5343 (14)
C2—H2B	0.9900	C8—H8A	0.9900
N3—C4	1.4696 (14)	C8—H8B	0.9900
N3—C10	1.4769 (12)	C9—H9A	0.9900
C4—N5	1.4733 (13)	C9—H9B	0.9900
C4—H4A	0.9900	C10—H10A	0.9900
C4—H4B	0.9900	C10—H10B	0.9900
N5—C9	1.4691 (14)	N11—H11A	0.898 (12)
N5—C6	1.4780 (13)	N11—H11B	0.895 (14)
C6—C7	1.5383 (14)		
			
C2—N1—C9	107.74 (8)	N11—C7—C10	109.53 (8)
C2—N1—C8	108.41 (8)	N11—C7—C8	111.06 (8)
C9—N1—C8	108.81 (8)	C10—C7—C8	107.12 (8)
N1—C2—N3	113.33 (8)	N11—C7—C6	113.78 (8)
N1—C2—H2A	108.9	C10—C7—C6	107.16 (8)
N3—C2—H2A	108.9	C8—C7—C6	107.92 (8)
N1—C2—H2B	108.9	N1—C8—C7	111.01 (8)
N3—C2—H2B	108.9	N1—C8—H8A	109.4
H2A—C2—H2B	107.7	C7—C8—H8A	109.4
C4—N3—C2	107.35 (8)	N1—C8—H8B	109.4
C4—N3—C10	108.97 (8)	C7—C8—H8B	109.4
C2—N3—C10	108.54 (8)	H8A—C8—H8B	108.0
N3—C4—N5	113.67 (8)	N5—C9—N1	113.80 (9)
N3—C4—H4A	108.8	N5—C9—H9A	108.8
N5—C4—H4A	108.8	N1—C9—H9A	108.8
N3—C4—H4B	108.8	N5—C9—H9B	108.8
N5—C4—H4B	108.8	N1—C9—H9B	108.8
H4A—C4—H4B	107.7	H9A—C9—H9B	107.7
C9—N5—C4	107.51 (9)	N3—C10—C7	110.95 (8)
C9—N5—C6	108.26 (8)	N3—C10—H10A	109.4
C4—N5—C6	107.99 (8)	C7—C10—H10A	109.5
N5—C6—C7	111.46 (8)	N3—C10—H10B	109.4
N5—C6—H6A	109.3	C7—C10—H10B	109.5
C7—C6—H6A	109.3	H10A—C10—H10B	108.0
N5—C6—H6B	109.3	C7—N11—H11A	112.4 (8)
C7—C6—H6B	109.3	C7—N11—H11B	107.6 (8)
H6A—C6—H6B	108.0	H11A—N11—H11B	110.9 (11)
			
C9—N1—C2—N3	57.51 (11)	C9—N1—C8—C7	−57.88 (10)
C8—N1—C2—N3	−60.09 (11)	N11—C7—C8—N1	−177.96 (8)
N1—C2—N3—C4	−57.78 (11)	C10—C7—C8—N1	−58.39 (10)
N1—C2—N3—C10	59.87 (11)	C6—C7—C8—N1	56.69 (10)
C2—N3—C4—N5	57.63 (11)	C4—N5—C9—N1	56.62 (11)
C10—N3—C4—N5	−59.73 (11)	C6—N5—C9—N1	−59.81 (11)
N3—C4—N5—C9	−57.06 (12)	C2—N1—C9—N5	−57.07 (11)
N3—C4—N5—C6	59.55 (12)	C8—N1—C9—N5	60.27 (11)
C9—N5—C6—C7	57.55 (11)	C4—N3—C10—C7	58.16 (11)
C4—N5—C6—C7	−58.57 (11)	C2—N3—C10—C7	−58.45 (11)
N5—C6—C7—N11	179.47 (8)	N11—C7—C10—N3	178.59 (8)
N5—C6—C7—C10	58.25 (10)	C8—C7—C10—N3	58.05 (10)
N5—C6—C7—C8	−56.81 (10)	C6—C7—C10—N3	−57.55 (10)
C2—N1—C8—C7	59.04 (11)		

### Hydrogen-bond geometry (Å, °)

**Table:**

*D*—H···*A*	*D*—H	H···*A*	*D*···*A*	*D*—H···*A*
N11—H11A···N3^i^	0.898 (12)	2.335 (13)	3.2316 (13)	176.0 (12)
N11—H11B···N11^ii^	0.895 (14)	2.253 (14)	3.1465 (14)	175.2 (11)

Symmetry codes: (i) *x*, *y*, *z*−1; (ii) −*y*+3/2, *x*, −*z*+1/2.
